# Review: Urinary Deposits: Their Diagnosis, Pathology, and Therapeutical Indications

**Published:** 1859-12

**Authors:** 


ART. VII.—*Urinary Deposits: their Diagnosis, Pathology, and Therapeutical Indications.* By GOLDING BIRD, M. D., F. R. S. Edited by EDMUND LLOYD BIRKETT, M. D., Caius Coll. Cantab., Fellow of the Royal College of Physicians; Physician to the City of London Hospital for Diseases of the Chest; formerly Curator of the Museum, and Secretary to the Clinical Society, Guy's Hospital; and Physician to the Surrey Dispensary. A new American, from the fifth London edition. With eighty illustrations on wood. Philadelphia: Blanchard & Lea, 1859.

ALL physicians who have attempted to keep up with the progress of medical knowledge, and apply to every-day practice the valuable truths with which science is daily enriched, are familiar with "Bird on Urinary Deposits." It has long been to the practitioner what "Wilson's Anatomy" is to the student, *the* work on the subject. We think all will agree with us when we say that "Bird" never disappoints us when we go to him for assistance in cases in practice; and will enable any one who has the use of a good microscope to become, in a short time, practically familiar with urinary deposits, and competent to make any ordinary examination of urine. The most severe test to which a work can be subjected is the one which the one before us has so long sustained; that is, the consultation of practitioners upon practical matters. A book may be elegantly written, every view enunciated with apparent clearness, and supported by convincing arguments; such a work may even be highly spoken of by the reviewers; but if it be unsound, and not based upon practical knowledge, its deficiencies will soon become known to the profession. Without referring to individual works, there are many which are good to read, but bad to study; which give a very favorable impression on the perusal of a few chapters, but which, when we desire practical information, are sadly deficient and unsatisfactory.

The importance to the practical physician of a knowledge of morbid urine, is so universally acknowledged that it is unnecessary to adduce any argument in its proof; this importance to practice is equaled by the interest of the subject to the investigator. Though we are now well acquainted with the composition of urinary deposits, and are able to recognize them with ease and certainty, yet a great deal remains to be done by investigation into the relations of these deposits to disease. We can, without the slightest difficulty, determine the presence of a phosphatic deposit, for example, in the urine; but are we able with the same certainty to give its relations to the disease which co-exists, or to determine, from an examination of the urine, what disease is likely to be present? This we are not yet able to do, and in this lies the tempting field for clinical investigators. Those who would be likely to use the work before us, are already acquainted sufficiently with its merit; and to them we have only to announce the appearance of a new edition. But there are some, and they are too numerous, who ignore, for some reason or another, all new things—some who will not, even now, avail themselves of the stethescope, the speculum, and other methods of physical diagnosis. Though we cannot ordinarily derive as much assistance from examination of the urine as we do from the stethescope or speculum, yet there are cases in which it is fully as important as either of these modes of investigation. We are confident that this disposition on the part of some members of the profession to ignore what others make use of with success, and what is sometimes, though rarely, indeed, made use of by unscrupulous quacks, does more to lower our standing as a profession with the people than any other one thing. Being unprovided with certain valuable means of recognizing disease, of course mistakes are made, and the individual loses, in some degree, the confidence of the public. Through him the profession suffers.

Any young man, with Bird on Urinary Deposits before him, and a good microscope at hand, may, if he take any pains to procure specimens of urine, be able, in a short time, to recognize the character of any deposit; and, as a practitioner, he will find the work as good as a consulting physician. The first edition which appeared in this country, was published in 1845, by Blanchard & Lea, and contained a little more than two hundred pages. The edition which is now presented to the profession is from the fifth which has been published in London. It has been much enlarged from the original, by the author, who is since dead, and further by the editor, Dr. Birkett, until it has reached 382 pages. We know of no more complete and reliable book on this subject; and as such, it will undoubtedly continue to receive the favor which it has hitherto enjoyed. Works upon practice of medicine and upon surgery are very well in their place, and will answer as mere text-books for students; but for one who wishes to make himself familiar with particular subjects, or who wishes, in any individual case, to get at the best and most reliable information upon any subject, monographs are necessary; for here we usually have the author speaking *ex cathedra;* while in the more comprehensive works, many subjects are necessary discussed in an imperfect manner from want of space, or because the author is not, himself, practically familiar with them.

